# Kinetics of the inhibition of renin and angiotensin I-converting enzyme by cod (*Gadus morhua*) protein hydrolysates and their antihypertensive effects in spontaneously hypertensive rats

**DOI:** 10.3402/fnr.v59.29788

**Published:** 2015-12-28

**Authors:** Abraham T. Girgih, Ifeanyi D. Nwachukwu, Fida Hasan, Tayo N. Fagbemi, Tom Gill, Rotimi E. Aluko

**Affiliations:** 1 Department of Human Nutritional Sciences, University of Manitoba, Winnipeg, Canada; 2 The Richardson Centre for Functional Foods and Nutraceuticals, University of Manitoba, Winnipeg, Canada; 3 Department of Process Engineering and Applied Science, Dalhousie University, Halifax, Canada; 4 Department of Food Science and Technology, Federal University of Technology, Akure, Nigeria

**Keywords:** cod, protein hydrolysate, angiotensin I-converting enzyme, renin, enzyme inhibition kinetics, IC_50_, systolic blood pressure, spontaneously hypertensive rats

## Abstract

**Background:**

Cod muscle has a balanced protein profile that contains potentially bioactive amino acid sequences. However, there is limited information on release of these peptides from the parent proteins and their ability to modulate mammalian blood pressure.

**Objective:**

The aim of this study was to generate cod antihypertensive peptides with potent *in vitro* inhibitory effects against angiotensin-converting enzyme (ACE) and renin. The most active peptides were then tested for systolic blood pressure (SBP)-reducing ability in spontaneously hypertensive rats (SHRs).

**Design:**

Cod protein hydrolysate (CPH) was produced by subjecting the muscle proteins to proteolysis first by pepsin and followed by trypsin+chymotrypsin combination. In order to enhance peptide activity, the CPH was subjected to reverse-phase (RP)-HPLC separation to yield four fractions (CF1, CF2, CF3, and CF4). The CPH and RP-HPLC fractions were each tested at 1 mg/mL for ability to inhibit *in vitro* ACE and renin activities. CPH and the most active RP-HPLC fraction (CF3) were then used for enzyme inhibition kinetics assays followed by oral administration (200 and 30 mg/kg body weight for CPH and CF3, respectively) to SHRs and SBP measurements within 24 h.

**Results:**

The CPH, CF3, and CF4 had similar ACE-inhibitory activities of 84, 85, and 87%, which were significantly (*p*<0.05) higher than the values for CF1 (69%) and CF2 (79%). Conversely, the CF3 had the highest (63%) renin-inhibitory activity (*p*<0.05) when compared to CPH (43%), CF1 (15%), and CF4 (44%). CPH and CF3 exhibited uncompetitive mode of ACE inhibition, whereas renin inhibition was non-competitive. Even at a 6.7-fold lower dosage, the CF3 significantly (*p*<0.05) reduced SBP (maximum −40.0 mmHg) better than CPH (maximum −19.1 mmHg).

**Conclusions:**

RP-HPLC fractionation led to enhanced antihypertensive effects of cod peptides, which may be due to a stronger renin-inhibitory activity.

Hypertension is related to elevated blood pressure (BP) and is an independent yet controllable risk factor for cardiovascular events such as myocardial infarction, atherosclerosis, stroke, and heart failure (1). It has been estimated by the World Health Organization that by 2020, heart disease and stroke will become the leading cause of death and disability globally (2). According to recent health reports, hypertension is considered a critical world health problem needing urgent attention and is estimated to affect up to 1.56 billion people by 2025 (3). In terms of actual numbers, hypertension accounts for approximately 17 million deaths a year, which is almost one-third of the total worldwide annual record of deaths (4). Physical activity and healthy diet could contribute to a significant reduction of about 30% in the morbidity and mortality that arise from hypertension (5). BP is controlled by the renin–angiotensin system in which renin produced from the kidney catalyzes the rate-limiting conversion of angiotensinogen to angiotensin I (AT-I), an inactive decapeptide (6). Angiotensin I-converting enzyme (ACE), which possesses a crucial role in BP regulation, then catalyzes the cleavage of the C-terminal His-Leu dipeptide of the resulting AT-I into angiotensin II, a potent octapeptide vasopressor. ACE also extends its catalytic impact towards the inactivation of bradykinin and kallidin, the vasodilator peptides (1). Under non-homeostatic or disease conditions, these catalytic actions of ACE can result in blood vessel wall stiffening and inability to relax following contraction, which, if not controlled, leads to hypertension. Synthetic ACE inhibitors (captopril, enalapril, lisinopril, etc.), renin inhibitor (aliskiren), and angiotensin receptor blockers are traditionally used to treat congestive heart failure and hypertension (7). However, there are reports of side effects such as cough, taste alterations, diarrhea, skin rashes, renal, and erectile dysfunction associated with these synthetic inhibitors which have resulted in an increased desire to find natural food-grade inhibitors (8–12). Natural food-based inhibitors that possess both renin- and ACE-inhibitory effects will usually be preferred as antihypertensive agents because this could provide more effective control of high BP, thus preventing hypertension. Currently, natural sources of renin and ACE inhibitors are being investigated as a milder but effective alternative for high BP control through protein hydrolysis using single strain or a mixture of enzymes or their sequential combination. To date, several food-grade peptide inhibitors with dual or multifunctional enzyme inhibitory properties from plant sources such as canola (13), rapeseed (14), and hemp seed (15) as well as animal sources such as chicken skin (16), cod (1), and catfish (17) have been produced. However, there is limited information on the mechanism of action and how chromatographic enrichment methods can be used to enhance potency of cod protein-derived peptides. Therefore, this work was based on the hypothesis that reverse-phase (RP)-HPLC separation of cod peptides can be used to isolate fractions with better *in vitro* and *in vivo* activities than the unfractionated protein hydrolysate. The aim of this work was to determine the renin- and ACE-inhibitory activities of Atlantic cod fish (*Gadus morhua*) enzymatic protein hydrolysate in comparison with RP-HPLC peptide fractions. The most active peptide RP-HPLC fraction and unfractionated protein hydrolysate were then used for comparative kinetic studies of renin- and ACE-inhibitory mechanisms as well as systolic blood pressure (SBP)-reducing ability in spontaneously hypertensive rats (SHRs).

## Materials and methods

### Materials

The Renin Inhibitor Screening Assay Kit was purchased from Cayman Chemicals (Ann Arbor, MI, USA). Enzeco Trypsin-Chymotrypsin^®^ 1:1 was obtained from Enzyme Development Corporation, New York, NY, USA. Pepsin, rabbit lung ACE, and *N*-[3-(2-furyl) acryloyl]-l-phenylalanyl-glycyl-glycine (FAPGG) were purchased from Sigma Chemicals (St. Louis, MO, USA). Methanol (HPLC grade), trifluoroacetic acid (TFA), 0.45- and 0.2-micron syringe filters, and all other analytical grade chemical reagents were obtained from Fisher Scientific (Oakville, ON, Canada).

### Preparation of cod protein hydrolysate

A detailed description of the cod muscle preparation and protein hydrolysis has been previously described (18). Briefly, 50 g of minced boneless cod muscle was suspended in 200 mL distilled water followed by homogenization in a standard Waring Blender for ~2 min at high speed. The homogenate was stored in the cold room overnight under constant stirring. The homogenate was then centrifuged at 7,000×*g* for 20 min at 4°C and the supernatant discarded while the pellet was collected and dispersed in 200 mL of distilled water. The protein dispersion was adjusted to pH 2.0 with 2 mol/L HCl and pepsin (600–1800 units/mg protein) added at 10,000 units/g fish protein. The enzyme-treated protein dispersion was incubated at 37°C for 12 h with continuous stirring and the digest adjusted to pH 7.8 with 2 mol/L NaOH to irreversibly deactivate pepsin. A trypsin-chymotrypsin (Enzeco Trypsin-Chymotrypsin 1:1) mixture was then added at 1,000 units per mg fish protein for both components and digestion allowed to proceed for 4 h at 37°C with constant stirring. The digest was then boiled for 10 min to inactivate the enzymes, cooled to room temperature, centrifuged (5,200×*g* for 30 min at 4°C), and the supernatant filtered through a Whatman #2 qualitative filter paper. The filtrate was passed through a 1 kDa molecular weight cut-off Prep/Scale Tangential Flow Filtration cartridge membrane ultrafiltration setup (Millipore Corporation, Bedford, MA, USA). The collected membrane permeate (<1 kDa) was lyophilized as the cod protein hydrolysate (CPH) and stored at −20°C.

### RP-HPLC separation of CPH

CPH was fractionated using RP-HPLC on a Varian 940-LC semi-preparative system according to a previously reported method (19). Briefly, the freeze-dried CPH was dissolved (100 mg/mL) in double-distilled water (DDW) that contained 0.1% TFA (solvent A) and 4 mL (sequentially filtered through 0.45 and 0.2 µm membrane disks) was injected onto a Phenomenex C12 preparative column (21×250 mm). Fractions were eluted from the column at a flow rate of 10 mL/min using a linear gradient of 0–100% solvent B (methanol that contained 0.1% TFA) over the course of 60 min. Peptide elution was monitored at 220 nm and eluted peptides collected using an automated fraction collector at every 1 min, which were then pooled into four fractions (CF1, CF2, CF3, and CF4) as previously reported (18). The pooled fractions were subjected to solvent evaporation in a rotary evaporator and the aqueous residues freeze-dried. Protein contents of CPH and its RP-HPLC peptide fractions were determined using the modified Lowry method (20).

### ACE inhibition assay

Ability of cod peptides to inhibit *in vitro* ACE activity was determined using a spectrophotometric method with FAPGG as substrate (21). Briefly, 1 mL of 0.5 mmol/L FAPGG (dissolved in 50 mmol/L Tris-HCl buffer containing 300 mmol/L NaCl, pH 7.5) was mixed with 20 µL ACE (1 U/mL, final activity of 20 mU) and 200 µL sample dissolved in same buffer as the FAPGG. The rate of absorbance decrease at 345 nm was recorded for 2 min at room temperature. The buffer was used instead of sample solutions in the blank experiment. ACE activity was expressed as rate of reaction (ΔA/min) and inhibitory activity was calculated as:

$$\text{ACE inhibition (}\%)=[1-\Delta A{\min^{-1}}_{\left(\mathrm{sample}\right)}/\Delta A{\min^{-1}}_{\left(\mathrm{blank}\right)}]\times 100$$

where Δ*A* min^-1^_(sample)_ and Δ*A* min^-1^_(blank)_ are ACE activity in the presence and absence of inhibitory peptides, respectively. The concentration of peptide that inhibited ACE activity by 50% (IC_50_) was calculated by non-linear regression from a plot of percentage ACE inhibition versus four peptide concentrations (0.125, 0.25, 0.5, and 1.0 mg/mL). The kinetics of ACE inhibition was studied with 0.0625, 0.125, 0.25, and 0.5 mmol/L substrate (FAPGG) concentrations. The mode of ACE inhibition was determined from the Lineweaver–Burk plots while kinetic parameters (*V*_max_ and *K*_m_) were estimated from non-linear regression fit of the data to the Michaelis–Menten equation using GraphPad Prism version 5.0 (GraphPad Software, San Diego, CA, USA). Inhibition constant (*K*_i_) was calculated as the x-axis intercept from a plot of the slope of the Lineweaver–Burk lines against sample concentrations while catalytic efficiency (CE) was calculated from *V*_max_/*K*_m_ ratio.

### Renin inhibition assay

*In vitro* assay of human recombinant renin activity was conducted using the Renin Inhibitor Screening Assay Kit according to the method previously described (22). Briefly, sample was diluted in Tris-HCl buffer (50 mmol/L, pH 8.0, containing 100 mmol/L NaCl), and prewarmed to 37°C prior to initiating the reaction. Before the reaction, 1) 20 µL substrate, 160 µL assay buffer, and 10 µL DDW were added to the background wells; 2) 20 µL substrate, 150 µL assay buffer, and 10 µL DDW were added to the control wells; and 3) 20 µL substrate, 150 µL assay buffer, and 10 µL sample were added to the inhibitor (sample) wells. The reaction was initiated by adding 10 µL renin to the control and sample wells. The microplate was shaken for 10 s for proper mixing and incubated at 37°C for 15 min; fluorescence intensity (FI) was then recorded at excitation and emission wavelengths of 340 and 490 nm, respectively, using a fluorometric microplate reader (Spectra MAX Gemini, Molecular Devices, Sunnyvale, CA, USA). The percentage renin inhibition was calculated as follows:

$$\text{Renin inhibition (}\%)=[1-\Delta \mathrm{FIU}{\min^{-1}}_{\left(\mathrm{sample}\right)}/\Delta \mathrm{FIU}{\min^{-1}}_{\left(\mathrm{blank}\right)}]\times 100$$

where Δ*FIU* min^-1^_(sample)_ and Δ*FIU* min^-1^_(blank)_ are renin activity in the presence and absence of inhibitory peptides, respectively. IC_50_ was calculated by non-linear regression from a plot of percentage renin inhibition versus peptide concentrations (0.125, 0.25, 0.5, and 1.0 mg/mL). The renin inhibition kinetics was conducted using 0.625, 1.25, 2.5, 5, and 10 µmol/L substrate concentrations in the absence and presence of samples, while kinetic parameters were calculated as described above for ACE.

### Evaluation of antihypertensive activity of cod peptides in SHRs

Animal experiments were carried out following the Canadian Council on Animal Care Ethics guidelines with a protocol approved by the University of Manitoba Animal Protocol and Management Review Committee. The male SHRs (Charles River Laboratories, Montreal, PQ) with 350–390 g body weight (bw) were kept in the Animal Housing Facility at the Richardson Centre for Functional Foods and Nutraceuticals, under a 12-h day and night cycle at 21°C and fed regular chow diet and tap water. The rats were divided into four groups (CPH, CF3, captopril, and phosphate-buffered saline, pH 7.4) with six rats per group. The CPH (200 mg/kg bw), CF3 (30 mg/kg bw), and captopril (10 mg/kg bw) were each dissolved in phosphate-buffered saline and administered to the SHRs by oral gavage followed by measurement of SBP over a 24-h period (at 2, 4, 6, 8, and 24 h) using the tail-cuff method in slightly anesthetized rats as previously described (22). Prior to oral gavage, the baseline (time zero) SBP was determined; the SBP changes (ΔSBP, mmHg) were determined by subtracting the baseline data from the values obtained at different time points.

### Statistical analysis

All assays were conducted in triplicate and analyzed by one-way analysis of variance (ANOVA). The means were compared using Duncan's multiple range test and significant differences accepted at *p*<0.05.

## Results

### Preparation of CPH and its RP-HPLC peptide fractions

Enzymatic hydrolysis of the cod fish muscles in this study using a combination of pepsin, trypsin, and chymotrypsin enzymes produced the CPH, which was further subjected to RP-HPLC fractionation to obtain four distinct peptide fractions namely CF1, CF2, CF3, and CF4 (21). The percent protein content of CPH and its RP-HPLC peptide fractions (CF1, CF2, CF3, and CF4) were determined to be 58, 40, 96, 91, and 94%, respectively.

### In vitro ACE and renin-inhibitory activities

**Fig. 1:**
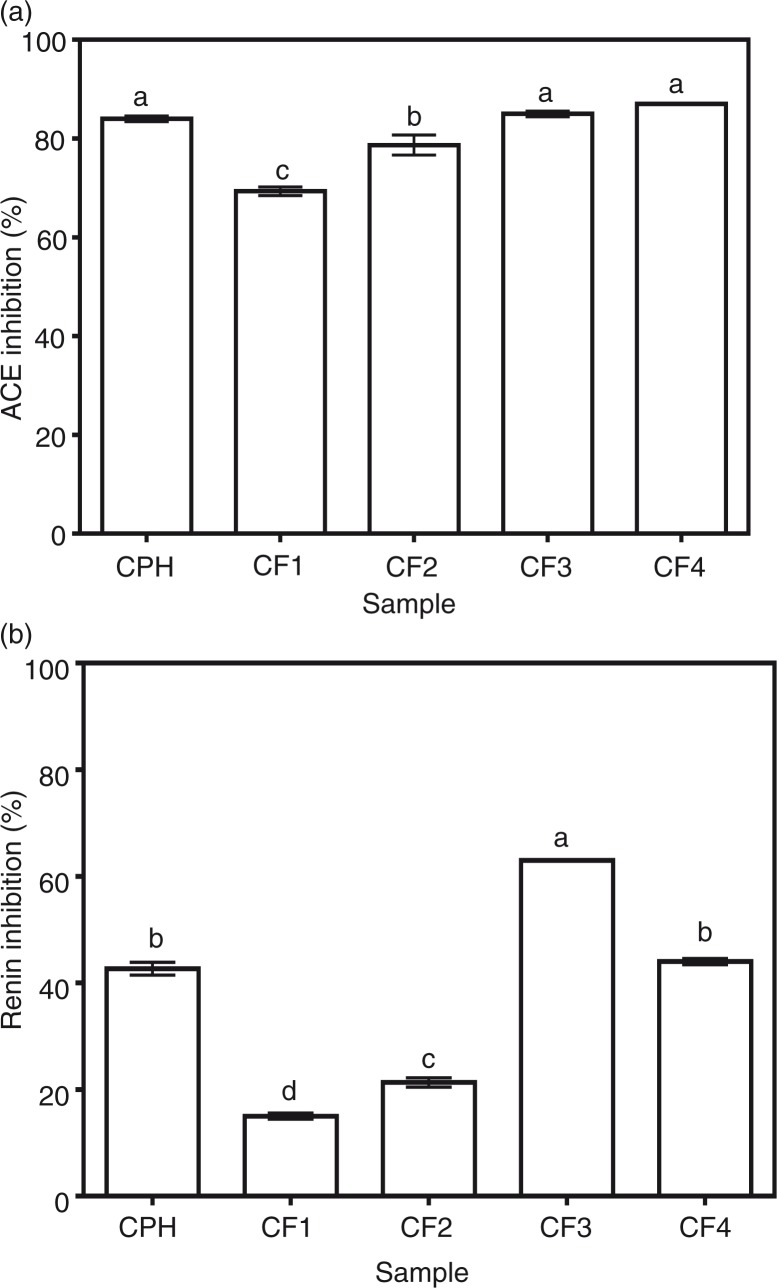
*In vitro* (a) angiotensin I-converting enzyme (ACE) and (b) renin-inhibitory activities of cod protein hydrolysate (CPH) and its RP-HPLC derived peptide fractions (CF1-CF4).

CPH and its RP-HPLC fractions (CF1, CF2, CF3, and CF4) were subjected to *in vitro* testing for ACE- and renin-inhibitory activities and the results obtained are shown in Fig. 1a and b. The CPH and its RP-HPLC-derived fractions had ACE-inhibitory activities which ranged in magnitude from 69 to 87% at the tested level of 1 mg/mL protein concentration. The less hydrophobic (most hydrophilic) RP-HPLC cod peptide fractions, CF1 (69%) and CF2 (79%) showed good abilities to inhibit ACE; however, these abilities were significantly (*p*<0.05) lower than the inhibitory levels exhibited by the most hydrophobic fractions CF3 (85%) and CF4 (87%). These high ACE activities of CF3 and CF4 are similar to the ACE-inhibitory activity of the CPH (84%).

**Fig. 2:**
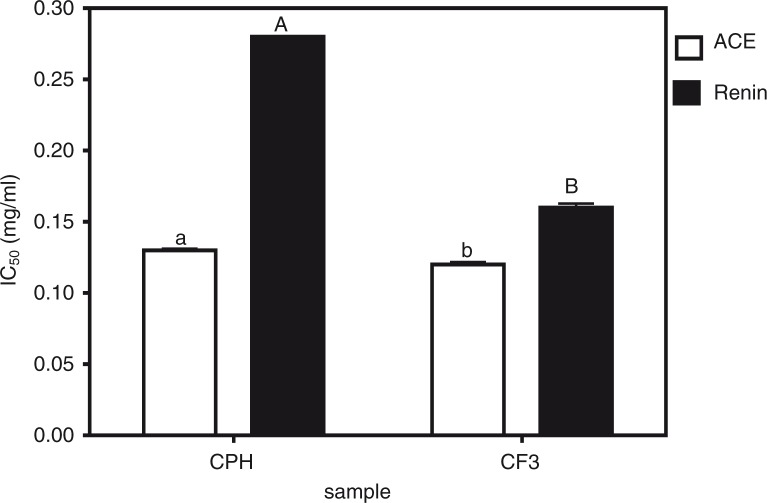
Peptide inhibitory concentrations of cod protein hydrolysate (CPH) and its most active RP-HPLC peptide fraction 3 (CF3) that reduced 50% of enzyme activity (IC_50_).

Figure 1b shows that the CF3 fraction had the highest while CF1 (15%) had the least *in vitro* renin-inhibitory activity. CPH (43% renin-inhibitory activity) fractionation significantly (*p*<0.05) improved renin-inhibitory activity only in CF3 but not in CF1, CF2, and CF4 which exhibited renin-inhibitory activities of 15, 21, and 44%, respectively. Peptide fraction hydrophobic character also seemed to play a role in renin inhibition just as observed for ACE inhibition. This is because the less hydrophobic CF1 and CF2 had lower renin-inhibitory values than the more hydrophobic CF3 and CF4. As expected, the renin inhibition potency of cod peptides (15–63%) was generally lower than that of ACE inhibition (69–87%). Based on the above *in vitro* bioactivity results, the most active fraction against both ACE and renin (CF3) was chosen for further comparison with CPH in terms of IC_50_ values, enzyme inhibition kinetics, and SBP-lowering ability in SHRs. The renin IC_50_ values as shown Fig. 2 confirms the significantly (*p*<0.05) stronger ACE- and renin-inhibitory properties of CF3 when compared to CPH.

### ACE-inhibitory kinetics of CPH and its RP-HPLC peptide fraction (CF3)

**Fig. 3:**
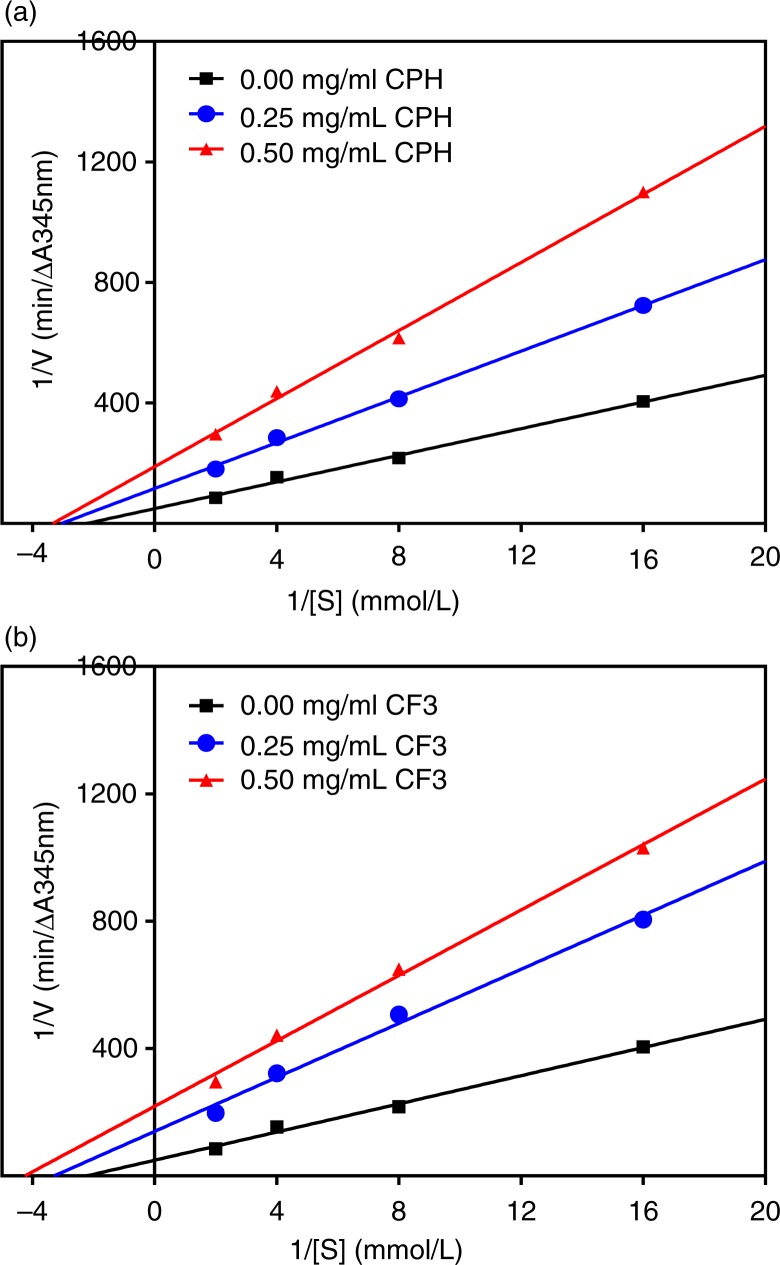
Lineweaver–Burk plots of angiotensin I-converting enzyme (ACE) inhibition at different peptide concentrations: (a) cod protein hydrolysate (CPH) and (b) most active RP-HPLC peptide fraction 3 (CF3).

In this study, we used Lineweaver–Burk plots to evaluate both the ACE and renin modes of inhibition of the most active CF3 fraction in comparison with the unfractionated hydrolysate (CPH). The double reciprocal plots for ACE-catalyzed reactions in the absence and presence of these cod peptides as shown in Fig. 3a and b for CPH and CF3, respectively, indicate a mostly uncompetitive type of inhibition. This is because the lines crossed each of the y- and x-axis at different points. Therefore, ACE-inhibitory *K*_m_ and *V*_max_ values for both CPH and CF3 were lower in the presence of peptides (Table 1). CE was also lower in the presence of CPH and CF3 peptides. The inhibition constant (*K*_i_), which is a measure of the inhibitor binding strength to ACE enzyme indicates that the CPH peptides (0.263 mg/mL) bind stronger than the CF3 peptides 0.394 mg/mL).

**Table 1:** Kinetics constants for angiotensin-converting enzyme (ACE) and renin inhibition by cod protein hydrolysate (CPH) and the most active RP-HPLC peptide fraction 3 (CF3)

Catalytic	ACE					Renin				
		
Parameter	CPH			CF3		CPH			CF3	
Peptide concentration (mg/mL)										
	0	0.25	0.5	0.25	0.5	0	0.3	0.6	0.25	0.5
*V*_max_	0.020±0.002	0.008±0.001	0.005±0.001	0.007±0.001	0.005±0.001	62.00±1.38	37.41±0.89	23.98±0.38	41.79±1.05	30.27±0.82
*K*_m_	0.449±0.012	0.327±0.020	0.299±0.009	0.304±0.016	0.235±0.011	5.214±0.124	4.995±0.110	4.800±0.105	4.789±0.152	4.680±0.107
CE	0.045±0.007	0.026±0.004	0.018±0.002	0.024±0.004	0.021±0.002	12.06±0.241	7.407±0.161	4.998±0.113	8.726±0.201	6.411±0.119
*K*_i_			0.263±0.011		0.394±0.017			3.294±0.105		2.152±0.005

*K*_m_, Michaelis–Menten constant (mmol/L); *V*_max_, maximum reaction velocity; CE, catalytic efficiency; *K*_i_, enzyme-inhibitor dissociation constant. The units are, respectively, Δ*A*/min and fluorescence intensity units/min for ACE and renin.

### Renin-inhibitory kinetics of CPH and CF3 RP-HPLC peptide fraction

**Fig. 4:**
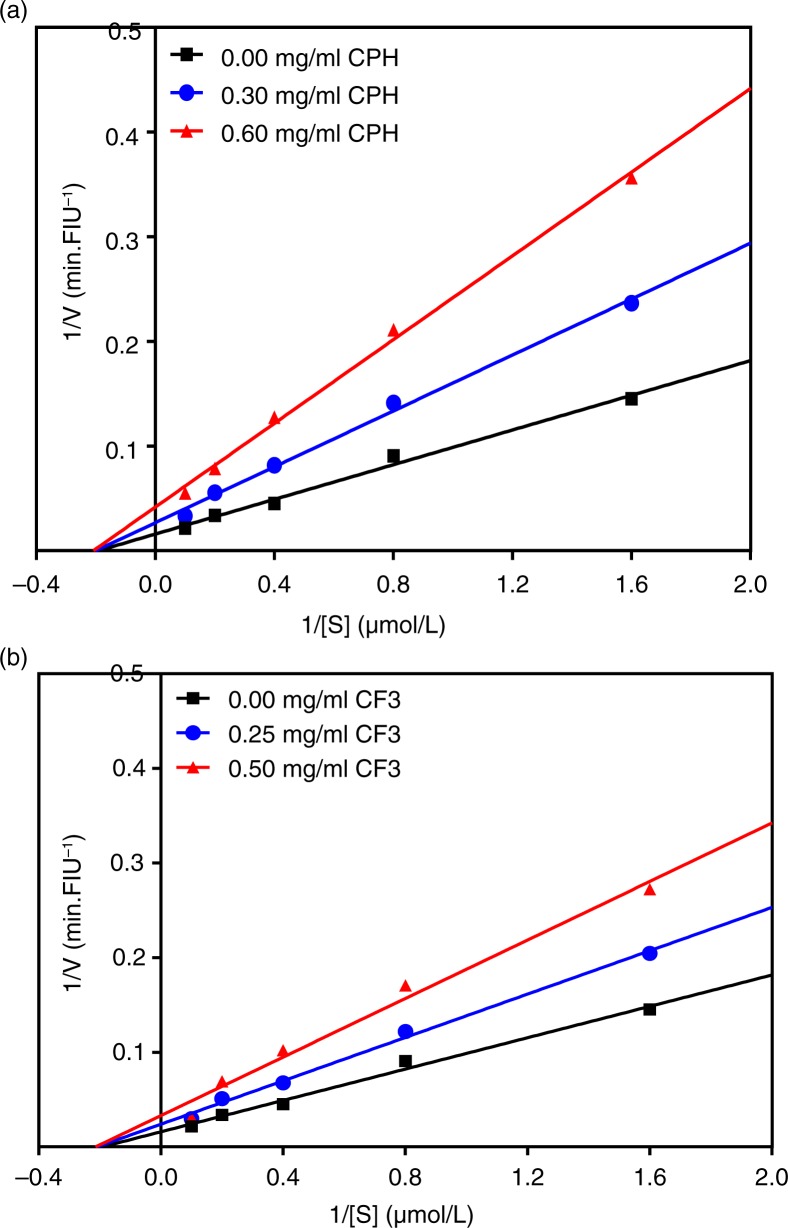
Lineweaver–Burk plots of human recombinant renin inhibition at different peptide concentrations: (a) cod protein hydrolysate (CPH) and (b) most active RP-HPLC peptide fraction 3 (CF3).

In contrast to ACE inhibition kinetics, Fig. 4a and b, respectively, showed that the CPH and CF3 exhibited a non-competitive mode of renin inhibition because the lines intersect the x-axis at approximately the same point. Non-competitive renin inhibition is typically exhibited with observed decreases in *V*_max_ but with similar *K*_m_ values (Table 1). The CE and *K*_i_ of the renin enzyme both decreased dose-dependently, suggesting that as the inhibitor concentration was increased the inhibitor binding affinity and inhibition efficiency for the enzyme was increased resulting in a reduction in the enzyme activity due to conformational changes.

### BP lowering effect of CPH and CF3 in SHRs

**Fig. 5:**
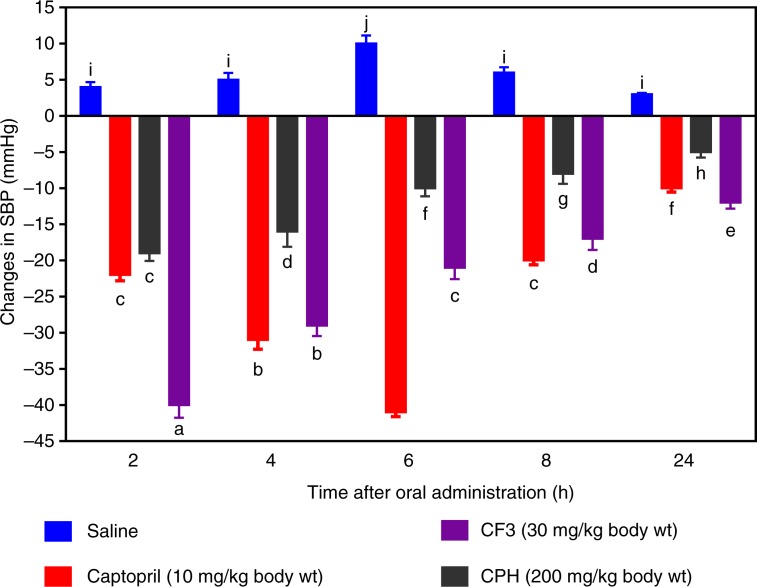
Time dependent changes in systolic blood pressure (SBP) of spontaneously hypertensive rats administered cod protein hydrolysate (CPH) and the most active RP-HPLC peptide fraction 3 (CF3).

In order to confirm the potential BP-reducing effects of cod peptides as observed during the *in vitro* tests, CPH and its most active RP-HPLC CF3 were orally administered to SHRs. As shown in Fig. 5, administration of CF3 (30 mg/kg bw) resulted in significant decreases in SBP at different time points when compared to saline (the negative control). The maximum SBP decrease due to CF3 was −40 mmHg after 2 h. Even though the hypotensive effect of CF3 gradually reduced with time, it was still significantly maintained up until 24 h (−12 mmHg) post-administration. Oral administration of CPH at a higher dose of 200 mg/kg bw led to a maximum SBP decrease of −19.1 mmHg also after 2 h, which is significantly (*p*<0.05) weaker than the 30 mg/kg bw lower dose of CF3. Moreover, persistence of the CPH was also weaker with a −5.0 mmHg effect after 24 h post-administration when compared to −12 mmHg for CF3.

## Discussion

### Protein content

The low protein content of the CPH may be attributed mainly to the presence of non-peptide components, especially salt, that were formed during proteolysis when NaOH was added to neutralize the protons released by the proteases. Therefore, for CF1, the low protein content can be attributed to co-elution of these salts with peptides. This is because the polar salts bind poorly to the hydrophobic reverse-phase column and will co-elute with early eluting peptides in CF1. The CF2–CF4 peptides that eluted later will contain less salt and more protein because most of the salts would have eluted with CF1. Similar results showing that the initial eluted fractions from hydrophobic chromatographic column exhibited low protein contents while later-eluting fractions had higher values have previously been reported for hemp seed protein hydrolysate fractions separated by RP-HPLC (23). Therefore, because peptide activity is the focus of this work, all experiments were conducted based on protein weight of the samples.

### ACE and renin inhibitions

Because all the samples contain a mixture of peptides, enzyme inhibitory activities of CPH and CF1–CF4 may perhaps be attributed to a synergistic effect of active cod peptides. In this respect, fractionation led to reduced peptide synergy in CF1 (69%) and CF2 (79%) because of the significantly (*p*<0.05) lower ACE and renin-inhibitory values when compared to CPH, CF3, and CF4. However, the reduced ACE and renin-inhibitory activities could also be due to interference from salts because CF1 and CF2 had lower protein contents and hence higher levels of non-peptide components. The results are similar to a previous work showing reduced ACE-inhibitory efficiency of rapeseed peptide fractions that had lower protein contents (14). For renin inhibition, fractionation effectively increased peptide inhibition only for CF3, which indicates the presence of peptides with strongest binding or synergistic activities. Thus, the ability of the cod peptides to inhibit both ACE and renin activities could be used as an effective tool to modulate the renin–angiotensin system in order to treat or prevent hypertension. Generally, ACE-inhibitory values were higher than renin-inhibitory values, which indicate that the cod peptides have stronger affinity for ACE. However, it should be noted that the two enzyme assay methods are different and direct comparison may not be possible. The renin- and ACE-inhibitory activities of cod peptides are in agreement with previous reports on food-based protein-derived peptides from different sources with multifunctional inhibitory activities against these vasoactive enzymes (14–16).

The IC_50_ values can also be used to evaluate potential potency and smaller values are preferred because the sample will be more effective at lower doses than samples with higher values. Therefore, the CF3 will be preferred as an inhibitory agent when compared to CPH based on their ACE and renin-inhibitory IC_50_ values. The CPH ACE-inhibitory IC_50_ value obtained in this work (0.13 mg/mL) is higher (weaker potency) than previously reported F3 permeates of skin (IC_50_=0.003 mg/mL) and bone (IC_50_=0.013 mg/mL) gelatins of *Pangasius* catfish (17), dried bonito (IC_50_=0.029 mg/mL) (24), and crude thermolysin hydrolysates from α-zein (IC_50_=0.021 mg/mL) (25). However, the CPH and CF3 have lower (stronger potency) ACE-inhibitory IC_50_ values than those reported for *ex vivo* myofibrillar digest and sarcoplasmic duodenal (IC_50_=1.06 and 2.16 mg/mL, respectively) (26), sea cucumber gelatin (IC_50_=0.35 mg/mL) (27), whole bovine casein (0.684 mg/mL) (28), and porcine (IC_50_=1.53 mg/mL) (29) protein hydrolysates. The CPH and CF3 also have lower ACE-inhibitory IC_50_ values than the F3-I (IC_50_=0.62 mg/mL) and F3-II (IC_50_=1.44 mg/mL) peptides of the second FPLC purification step of alcalase-derived seaweed pipefish muscle protein hydrolysate (30). In general the ACE-inhibitory IC_50_ values for food protein peptides are higher (lower potency) than the 0.00039–0.0085 mg/mL range reported for captopril, the antihypertensive drug (31). There are no available renin IC_50_ values for fish or marine peptides in literature to make fair comparison with our current results for cod peptides in this study. However, the renin-inhibitory IC_50_ values for CPH (0.28 mg/mL) and CF3 (0.16 mg/mL) are lower than the 0.81–1.89 mg/mL and 1.4–2.7 mg/mL values reported for hemp seed (22) and flaxseed (32) protein hydrolysates, respectively. The observed differences in IC_50_ values could be due to variations in peptide components, which would dictate strength of the synergistic interactions that are responsible for enzyme inhibition. Similar to the present results, the renin-inhibitory IC_50_ values obtained for chicken skin protein hydrolysates were also found to be higher than the ACE-inhibitory IC_50_ values (16), which supports literature evidence indicating renin as a more difficult enzyme to inhibit in comparison to ACE.

### Enzyme inhibition kinetics

It is vital to study the inhibitory kinetic properties of ACE and renin inhibitors because this helps reveal the possible mechanisms by which peptides are able to act as antihypertensive agents. ACE inhibition by CPH and CF3 was through uncompetitive mode, which means that the peptides were bound to the enzyme–substrate complex to form inhibitory complexes with reduced capacity to yield products. As evident in Table 1, binding of CPH and CF3 to ACE led to catalytic activity reduction, which is manifested as *V*_max_ decreases in proportion to peptide concentrations. The decreased ACE catalytic activity (or catalytic efficiency) in the presence of these peptides could be attributed to enzyme conformational rearrangement that reduced ability of the active site to accommodate the substrate. The *K*_m_ value in absence of an inhibitor (0.449 mmol/L FAPGG) in this study is slightly higher than the previously reported value of 0.306 mmol/L for flaxseed protein hydrolysate (32), but lower than the 0.6639 mmol/L for hemp seed protein hydrolysate (22). *K*_i_ is a measure of peptide affinity for the target enzyme and lower values indicate stronger binding potential than higher values. Table 1 shows higher ACE-binding ability by CPH than CF3 with lower and higher *K*_i_ values, respectively, though this trend did not translate to better inhibition by CPH. However, the *K*_i_ values for CPH and CF3 recorded in this study are lower than some of the values (up to 4.74 mg/mL) reported for the hemp seed protein hydrolysate fractions (22). In contrast, the *K*_i_ values are higher than the 0.044–0.051 mg/mL reported for chicken skin protein hydrolysate and peptide fractions (33).

Contrary to the ACE inhibition pattern, the CPH and CF3 inhibited renin activity mostly through a non-competitive mode. This implies that the inhibitor can achieve its inhibitory effects by binding to both the free renin and renin–substrate complex. In either case the enzyme–peptide interactions reduce enzyme affinity for substrate and hence less conversion to products as evident by the decreased *V*_max_ values shown in Table 1. The non-competitive mechanism of renin inhibition by cod peptides obtained in this study is different from the uncompetitive and mixed-type modes reported for flaxseed cationic (32) and hemp seed protein hydrolysate peptides, respectively (22).

### Antihypertensive effects in SHRs

The CF3 peptide demonstrated an excellent potential antihypertensive agent because of the fast-acting BP-lowering effect in SHRs within the first 2 h after oral administration and the persistent effect observed after 24 h. The results are consistent with the higher renin-inhibitory activity of CF3 when compared to CPH (Figs. 1b and 2). On a weight basis, the CF3 was about 12 times as potent as the CPH in BP-reducing potency. The results suggest that CPH and CF3 peptides were absorbed into the blood from the SHR gastrointestinal tract but the CF3 may have been absorbed more quickly. It is also possible that the CF3 peptides had stronger inhibitory effects during *in vivo* interactions with ACE and renin. The persistent BP-reducing effect of CF3 suggests that the peptides were perhaps more resistant to *in vivo* proteases than the CPH peptides. It is difficult to compare the CF3 data with literature values because of the differences in dosage administered to the SHR. For example, 10 and 100 mg/kg bw bovine casein hydrolysate produced −28 and −42 mmHg SBP reductions, respectively, 2 h after oral administration to SHRs (34). In contrast, a peptic oyster digest had weaker BP-reducing effects with −16 mmHg reduction after 4 h (35). Translating the 30 mg/kg bw CF3 dose which caused a −40.0 mmHg BP reduction in the SHRs indicates a potentially effective dose of only 350 mg/day for a 70 kg human being (36). Because the protein hydrolysate was obtained using pepsin and trypsin digestions, the nutritional implication could mean potential cardiovascular health benefits of cod protein consumption. This is supported by the work of Yahia et al. (37) who reported that addition of 20% (w/w) unhydrolyzed fish protein to the diet resulted in −31 mmHg reduction in SHR SBP after a 2-month feeding study. However, because the source of fish protein was not indicated, direct comparison with our current work is not possible.

In conclusion, although *in vitro* assays provide rapid assessment of bioactive potential of peptides, the use of physiologically relevant animal disease models provides for a realistic estimation of applicability to human disease conditions. Hence, the positive relationship between *in vitro* and *in vivo* data in this work provides a scientific basis to use these peptides in future human intervention trials to determine potential therapeutic utility. Although the expected human dose will be several times more than that of a drug, the lack of substantial negative side effects during peptide use as reported by several researchers should make the cod peptides attractive antihypertensive options.
